# Genome-Wide Identification of SNARE Family Genes and Functional Characterization of an R-SNARE Gene *BbSEC22* in a Fungal Insect Pathogen *Beauveria bassiana*

**DOI:** 10.3390/jof10060393

**Published:** 2024-05-31

**Authors:** Fang Li, Juefeng Zhang, Haiying Zhong, Kaili Yu, Jianming Chen

**Affiliations:** Institute of Plant Protection and Microbiology, Zhejiang Academy of Agricultural Sciences, Hangzhou 310021, China; lifang870910@163.com (F.L.);

**Keywords:** *Beauveria bassiana*, SNAREs, vesicular trafficking, fungal growth, stress tolerance, virulence

## Abstract

Soluble N-ethylmaleimide-sensitive factor attachment protein receptors (SNAREs) are central components of the machinery mediating cell membrane fusion and intracellular vesicular trafficking in eukaryotic cells, and have been well-documented to play critical roles in growth, development, and pathogenesis in the filamentous fungal plant pathogens. However, little is known about the contributions of SNAREs to the physiology and biocontrol potential in entomopathogenic filamentous fungi. Here, a genome-wide analysis of SNARE genes was performed taking advantage of the available whole genome sequence of *Beauveria bassiana*, a classical entomopathogenic fungus. Based on the compared genomic method, 22 genes encoding putative SNAREs were identified from the whole genome of *B. bassiana*, and were classified into four groups (7 Qa-, 4 Qb-, 6 Qc-, and 5 R-SNAREs) according to the conserved structural features of their encoding proteins. An R-SNARE encoding gene *BbSEC22* was further functionally characterized by gene disruption and complementation. The *BbSEC22* null mutant showed a fluffy appearance in mycelial growth and an obvious lag in conidial germination. The null mutant also exhibited significantly increased sensitivity to oxidative stress and cell wall perturbing agents and reduced the yield of conidia production by 43.1% compared with the wild-type strain. Moreover, disruption of *BbSEC22* caused a significant decrease in conidial virulence to *Spodoptera litura* larvae. Overall, our results provide an overview of vesicle trafficking in *B. bassiana* and revealed that BbSec22 was a multifunctional protein associated with mycelial growth, sporulation, conidial germination, stress tolerance, and insecticidal virulence.

## 1. Introduction

The soluble N-ethylmaleimide-sensitive factor attachment protein receptors (SNAREs) are important components of the vesicle trafficking machinery in eukaryotic cells, which is essential for many cellular processes including polarized growth and secretion of extracellular proteins in fungal organisms [1,2,3]. SNAREs form a superfamily of small proteins in yeast and filamentous fungi, for instance, there are 24 members in *Saccharomyces cerevisiae*, 21 in both *Aspergillus oryzae* and *Fusarium graminearum*, 35 in *Phytophthora sojae* and 22 in *Verticillium dahlia* [4,5,6,7,8]. Despite their differences in sizes among different organisms, the SNAREs are structurally characterized by a conserved segment of approximately 60–70 amino acids arranged in heptad repeats, termed the SNARE domain at the C-terminus [9,10]. In the process of vesicle trafficking, SNAREs anchored on different membranes interact through their SNARE domains to form a four-helix SNARE bundle which leads to a tight connection of the membranes that are destined to fuse and initiate the membrane merger [11]. Originally, SNAREs were functionally classified into v-SNAREs and t-SNAREs based on their localization to either vesicle membranes or target membranes [12]. However, many SNAREs were later found on both vesicle and target membranes, and SNAREs were reclassified into Q-SNAREs and R-SNAREs according to their sequence homology and the presence of an arginine (R) or glutamine (Q) residue at the zero layer in the four-helical bundles of SNARE domain [13]. In most cases, R-SNAREs act as v-SNAREs and Q-SNAREs act as t-SNAREs [14].

SNAREs-mediated vesicle trafficking was traditionally viewed as a housekeeping process and SNAREs have been well characterized in mammals, plants, and yeasts [15,16,17]. Excepting for the vital roles in membrane trafficking, recent findings in fungal cells show that SNAREs also play diverse roles in fungal growth and pathogenicity as well as asexual and sexual reproduction [18,19,20]. In *Neurospora crassa*, SNARE nsyn1 was found to be important for asexual conidiation and male mating, while SNARE nyn2 was revealed to be essential for hyphal branching and ascospore development [21]. Disruption of the orthologous genes (*GzSYN1* and *GzSYN2*) in the plant pathogen fungus *Gibberella zeae* also seriously reduced hyphal extension and eliminated female fertility [22]. In *Fusarium verticillioides*, the *FvSYN1* deletion mutant exhibited rough and hyper-branched, increased sensitivity to cell wall stress, and decreased conidial virulence [23]. Functionally characterization of several SNARE-encoding genes including *MoSSO1*, *MoSYN8*, *MoTLG2*, *MoVAM7*, and *MoSEC22* in the rice blast fungus *Magnaporthe oryzae* revealed that SNAREs were not only essential for fungal growth, conidiation, and virulence, but also involved in stress tolerance and cell wall integrity maintenance [24,25,26,27,28]. The R-SNARE Sec22 was required for vegetative growth, pathogenicity, and deoxynivalenol (DON) toxin production in the wheat scab fungus *F. graminearum* [29]. Deletion of a Sec22 orthologous VdSec22 in the vascular wilt fungus *V. dahlia* resulted in reduced virulence and suppressed secretion of carbohydrate hydrolase [30]. Recent studies also reported that SNAREs (DdVam7 and AoSec22) were determinant to hyphal morphogenesis, sporulation capacity, vacuole assembly, and trap formation in the nematode-trapping fungi *Drechslerella dactyloides* and *Arthrobotrys oligospora* [19,31]. Although the SNAREs have been extensively investigated in budding yeast, phytopathogenic fungi, and nematode-trapping fungi, no effort has been made to investigate the roles of SNARE orthologs in the mediation of fungal growth, sporulation, virulence, and multi-stress tolerance in insect fungal pathogens.

*Beauveria bassiana* is one of the classic entomopathogenic fungi that has been developed as successful mycoinsecticides for insect pest control [32,33]. However, their slow kill speed and poor tolerance to adverse environmental factors have restrained their application and commercial development [34]. Since SNAREs are highly conserved among fungi and functionally versatile, it is essential to elucidate their contributions to the physiology and biocontrol potential for the insect pathogen. This study sought to identify the SNARE superfamily genes in whole genome sequences of *B. bassiana*. Moreover, we choose an R-SNARE *BbSEC22*, an ortholog of *S. cerevisiae SEC22*, for further functional characterization by constructing the disruption and complementation mutants.

## 2. Materials and Methods

### 2.1. Microbial Strains and Culture Conditions

The wild-type strain *B. bassiana* (CICC 41021) was cultured on the plates of Sabouraud dextrose agar plus 1% yeast extract (SDAY) (Solarbio, Beijing, China) at the regime of 25 °C and 12:12 h (light/dark cycle). *Escherichia coli* DH5α were cultured in Luria–Bertani medium (Solarbio, Beijing, China) at 37 °C and used for vector construction. *Agrobacterium tumefaciens* AGL-1 used for fungal transformation was cultured at 28 °C in YEB medium (Solarbio, Beijing, China).

### 2.2. Identification and Analysis of Predicted SNAREs Encoding Genes

Amino acid sequences of SNAREs in *S. cerevisiae*, *A. oryzae*, and *P. sojae* obtained from the GenBank database were used as queries to search for putative SNAREs-encoding genes in the complete genome of *B. bassiana* (GenBank Accession Number: ADAH00000000) via tBLASTN analysis [35]. The amino acid sequences of predicted *B. bassiana* proteins showing significant similarity to SNARE queries and their open-reading frame sequences were extracted. These sequences were subjected to conserved domain and motif analysis using the online tools CD-Search (https://www.ncbi.nlm.nih.gov/Structure/cdd/wrpsb.cgi, accessed on 5 January 2024), SMART (http://smart.embl-heidelberg.de/, accessed on 5 January 2024) and InterProScan (http://www.ebi.ac.uk/interpro/search/sequence/, accessed on 5 January 2024). For phylogenetic analysis of a conserved R-SNARE protein BbSec22, the Sec22 homologs in other fungi were downloaded from the GenBank database via BLASTp (https://blast.ncbi.nlm.nih.gov/Blast.cgi, accessed on 5 January 2024). Protein sequence alignment was carried out using the software ClustalW (https://www.genome.jp/tools-bin/clustalw, accessed on 10 January 2024) with default settings [36]. The MEGA X software (https://www.megasoftware.net/, accessed on 10 January 2024) was applied to construct a phylogenetic tree using the neighbor-joining method with a bootstrap test of 1000 replicates [37].

### 2.3. Targeted Gene Deletion and Complement of BbSEC22

A conserved R-SNARE encoding gene *BbSEC22* was disrupted from *B. bassiana* using an *Agrobacterium*-mediated homologous recombination method. The plasmids p0380-bar and p0380-sur-gateway were used as backbones to delete and complement the target gene [38]. Briefly, two fragments with the length of 2071 bp and 2284 bp corresponding to the 5′ and 3′ flanking regions of *BbSEC22* were amplified by conventional PCR with pair primers listed in Table S1, respectively. The upstream and downstream fragments of *BbSEC22* were separately digested by two pairs of restriction enzymes *Hin*dIII/*Sac*I and *Bgl*II/*Spe*I (New England Biolabs, Hitchin, UK), and then successively cloned into the corresponding sites of the plasmid p0380-bar, which vectoring phosphinothricin (PPT) resistance gene *BAR*, the resultant plasmid p0380-bar-BbSec22 was used for target-gene disruption.

To construct plasmids for gene complementation, a 5197-bp PCR fragment containing the full-length *BbSEC22* coding region, the 2519-bp upstream region, and the 1982-bp downstream regions was amplified from *B. bassiana* genomic DNA and ligated into p0380-sur-gateway to replace the gateway fragment. The new plasmid p0380-sur-BbSec22 vectoring the second marker *SUR* (*M. grisea* acetolactate synthase gene resisting sulfonylurea) was used for target gene complementation.

The plasmids p0380-bar-BbSec22 and p0380-sur-BbSec22 were separately transformed into *A. tumefaciens* AGL-1 for transformation into the wild-type strain and the corresponding deletion mutant, respectively. The mutants of Δ*BbSEC22* and Δ*BbSEC22/BbSEC22* were selected from Czapek’s plates containing PPT (200 µg mL^−1^) or chorimuron ethyl ammonium (10 µg mL^−1^) and identified by PCR, reverse transcription PCR (RT-PCR) and quantitative real-time PCR (qRT-PCR) with primers listed in Table S1.

### 2.4. qRT-PCR Analysis of Gene Expressions

The temporal transcript patterns of *BbSEC22* in the wild-type cultures were assessed during 8-day growth at 25 °C and 12:12 h on cellophane overlaid on SDAY plates, on each of which 100 μL suspension with the concentration of 1 × 10^7^ conidia mL^−1^ (the same below unless mentioned otherwise) was spread to initiate the cultures. Total RNA was separately extracted using TRIzol^TM^ Plus Reagent (Takara, Shiga, Japan) and treated with DNase I (New England Biolabs, Hitchin, UK) following the manufacturer’s instructions. Every 5-μg RNA sample was reversely transcribed with a PrimeScript^TM^ RT reagent Kit (Takara, Tokyo, Japan). The transcript levels of *BbSEC22* and *18S rRNA* (used as internal standard) were assessed in the triplicate assays of qRT-PCR with Sec22q-F/R and 18S-F/R (Table S1). All qRT-PCR reactions were performed using an ABI Prism 7500 system (Thermo Fisher Scientific, Waltham, MA, USA) with SYBR^®^ Premix Ex Taq™ II (Takara, Japan). The relative expression level of *BbSEC22* was estimated as the transcript ratio of a given culture day over the first day using the 2^−ΔΔCt^ method [39].

For assaying the expression level alteration of selected genes caused by *BbSEC22* disruption, aliquots of 100 μL suspension of the wild-type strain and each mutant strain were spread onto the cellophane-attached plates of SDAY, followed by incubation of 3 days at the same regime. The selected genes were those possibly involved in fungal conidiation and responses to the stresses of oxidation and cell wall disturbance. Total RNA was extracted, transcribed, and subjected to qRT-PCR analysis with paired primers (Table S2) using the same protocol as described above. Relative expression levels of target genes were obtained by normalization to the transcript level of *18S rRNA*. The transcript levels of the other four R-type SNARE genes (*BbYKT6*, *BbNYV1*, *BbNYV2*, and *BbSNC1*) in the wild-type strain, deletion, and complementation mutants grown for 3 days on SDAY at 25 °C were also assessed using the same protocol.

### 2.5. Assays for Vegetative Growth, Sporulation and Conidial Germination

To assess the effect of *BbSEC22* disruption on the fungal mycelial growth, 1 µL conidial suspensions of the wild-type strain or each mutant were spotted centrally onto SDAY plates (9-cm diameter) and incubated at 25 °C. The diameter of each colony was then measured after incubation for 8 days. For biomass quantification, each of 100 µL conidial suspensions were pipeted into 100 mL SDB media (SDB: agar-free SDAY) and shaken at 25 °C and 160 rpm for 3 days, mycelia were then harvested by centrifugation, lyophilized overnight and weighed. For hyphal morphology examination, the mycelia of each strain were harvested after incubating in SDB media for 3 days and observed separately under a microscope. The cell wall and hyphal septum were visualized by calcofluor white (CFW) staining as described [40].

To assess the effect of *BbSEC22* on conidium production, 100 μL conidial suspension of each strain was spread onto cellophane-attached SDAY plates, followed by 8-day incubation at 25 °C. Conidial yield was estimated as of the number of conidia/cm^2^ colony by washing off conidia into 1 mL of 0.02% Tween 80 from each of three colony discs (5-mm diameter) through supersonic vibration and determining conidial concentration in the suspension using microscopic counts with a hemocytometer.

To assess the effect of *BbSEC22* on conidial germination, 50 µL conidial suspension of each strain was spread onto the plates of germination medium (GM: 2% sucrose and 0.5% peptone and 1.5% agar), followed by 24 h incubation at 25 °C and 12:12 h. From 6 h onwards, percent germination on each plate was assessed every 2 h using three microscopic counts (>100 conidia per count). All the above experiments were repeated three times each with three replicates.

### 2.6. Assays for Stress Tolerance of the Growing Colonies and Germling Conidia

To assess how of *BbSEC22* deletion affected resistance to environmental stresses, fungal mass discs (5-mm diameter) were cut from the colony edge of each strain grown for 3 days on cellophane-attached SDAY plates at 25 °C and transferred onto the center of one-quarter SDAY plates alone (control) or supplemented with one of the stressful chemicals (treatments) as follows: hyper-oxidative agents (40 mM H_2_O_2_ and 3 mM menadione), hyper-osmotic agents (1 M of NaCl and KCl), cell wall biosynthesis inhibitors (0.1 mg mL^−1^ SDS, 2 mg mL^−1^ congo red and 5 mg mL^−1^ CFW) and fungicides (1 μg mL^−1^ carbendazim and 0.1 mg mL^−1^ dimetachlone). All the plates were incubated for 7 days at the same regime and the resultant colonies were cross-measured for their diameters (mm). For each strain, relative growth inhibition was calculated as (C − N)/(C − 5) × 100, where C is the control (not stressed) colony diameter and N is the colony diameter under a given stress.

Apart from the assays of colony responses, conidia were quantitatively assayed for their tolerances to thermal stress using the same method described in a previous study [41]. Briefly, each 1 mL conidial suspension in glass tubes was exposed to the wet-heat stress of 45 °C for up to 90 min. Aliquots with a volume of 100 µL from each tube were separately pipetted every 15 min and released into 1 mL germination broth (GB: agar-free GM). After 24 h incubation at 25 °C, percent germinations were determined with the microscopic counts, and median lethal times (LT_50_) were then estimated by the probit analysis using the software GraphPad Prism 8.

### 2.7. Assays for Fungal Virulence

The wild type and mutant strains were bioassayed for their virulence to the second-instar larvae of *Spodoptera litura* using a lotus leaf disc system [38]. In brief, aerial conidia from a 7-day growth fungal culture on SDAY plates of each strain were harvested by washing with 2 mL distilled water with 0.02% Tween 80 and adjusted to a final concentration of 1 × 10^8^ conidia mL^−1^. Batches of 35–40 larvae on lotus leaf discs (~10 cm diameter) were separately sprayed with an equal volume (1 mL) of conidial suspension or 0.02% Tween 80 (control) using a handheld micro sprayer. After spray treatment, all larvae were reared on the leaf discs in Petri dishes for 7 days at 25 °C and 12:12 h and examined daily for mortality records. Fresh leaf discs were supplied daily for their feeding during the period. Each treatment was replicated three times. The fitted time-mortality relationship was used to estimate the LT_50_ values of each fungal strain against *S. litura* larvae.

### 2.8. Statistical Analysis

DPS software 7.05 was used for statistical analysis [42]. All data from the repeated experiments were expressed as mean ± standard error. Significant differences between means were tested using one-way analysis of variance (ANOVA) with Tukey’s HSD post-hoc analysis. Differences were considered to be significant at *p* < 0.05.

## 3. Results

### 3.1. Identification of SNARE Family Genes in B. bassiana Genome

**Figure 1:**
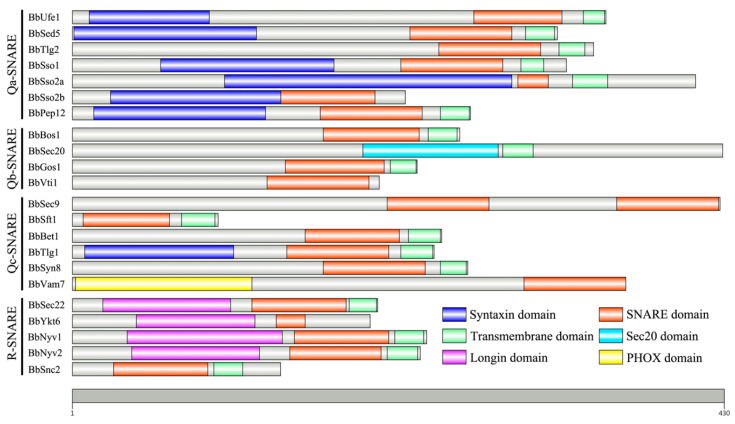
Structural schemes of SNARE proteins of *B. bassiana*. Types and names of proteins are listed on the left. Domain structures are drawn to represent their relative positions along the protein chain. The grey bar below represents the length scale of the protein sequences.

A total of 22 SNAREs were identified from the *B. bassiana* genome based on tBLASTN searches with representative SNAREs from well-characterized model organisms (Table 1). Each of them has one counterpart protein in *S. cerevisiae*, except for an unconventional SNARE-encoding protein Use1 which was not found ortholog in the *B. bassiana* genome. Additionally, *B. bassiana* possesses two orthologs for Sso2 while *A. oryzae* and *S. cerevisiae* have only one. In general, the deduced SNAREs share much higher identities when the entire amino acid sequences were compared with the SNAREs from *A. oryzae* than that from *S. cerevisiae*. Conserved domain analysis revealed that all of them have typical characteristic features of SNARE family proteins. One SNARE core motif and a transmembrane domain are generally conserved at the C-termini of each protein, although five SNAREs (BbSso2b, BbVti1, BbVam7, BbSec9, and BbYkt6) possess no transmembrane regions and one SNARE (BbSec20) have no SNARE motif instead of Sec20 motif. Two SNARE motifs existed in the BbSec9 sequence and one PHOX motif was presented in the N-terminus of BbVam7. Based on the highly conserved residues, the 22 SNAREs from *B. bassiana* were divided into Q- and R-types, and Q-types were further divided into three sub-types: Qa, Qb, and Qc (Figure 1). Remarkably, the glutamine residue (Q) conserved among Q-type SNAREs is replaced by an unconventional amino acid residue in three Qc-type SNAREs, that is an aspartic acid (D) in BbSft1, a histidine (H) in BbSyn8 and a serine (S) in BbBet1, respectively.

### 3.2. Structural and Expression Features of BbSec22

**Figure 2:**
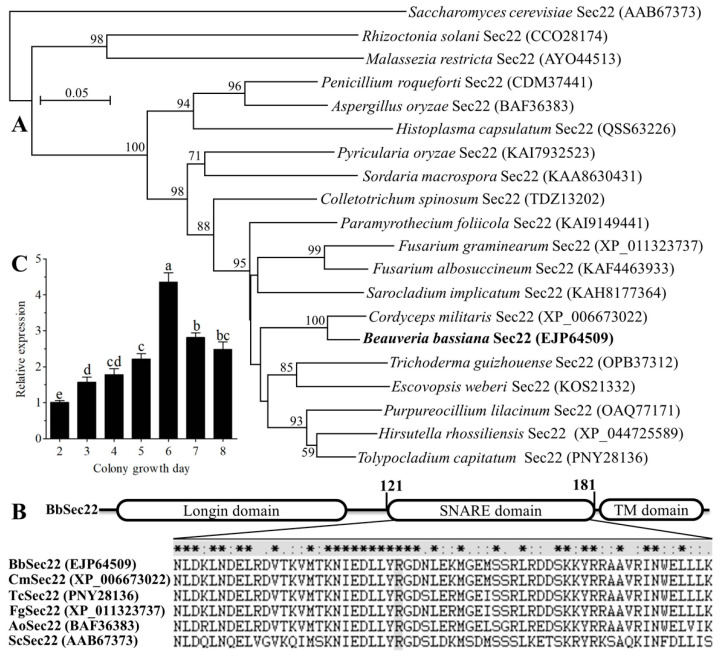
Phylogenetic, structural, and expressional features of BbSec22 of *B. bassiana*. (**A**) Phylogenetic comparison of BbSec22 with other fungal Sec22 proteins in the GenBank database. The bootstrap values are given at nodes. The GenBank accession number of a given Sec22 protein is parenthesized following the name of each fungal species. *B. bassiana* BbSec22 is shown in bold. (**B**) Schematic structure of BbSec22 protein and alignment of the six conserved SNARE domains from *Cordyceps militaris* (Cm), *Tolypocladium capitatum* (Tc), *Fusarium graminearum* (Fg), *Aspergillus oryzae* (Ao) and *Saccharomyces cerevisiae* (Sc). The closed triangle indicates the conserved zero-layer residue (Arginine, R) in the four-helical bundles of the SNARE domain. Invariant residues are asterisked while colons or periods denote conservative replacements. (**C**) Relative transcript levels of the *BbSEC22* gene during the 8-day incubation of *B. bassiana* on SDAY plates at 25 °C. Error bars: standard deviation of the mean from three replicates. The different letters above bars denote significant difference (Tukey’s HSD, *p* < 0.05).

The gene encoding an R-SNARE (BbSec22) was obtained by PCR amplification and introns were confirmed by RT-PCR. A full-length sequence of the *BbSEC22* gene consisted of a 696-bp ORF and an 87-bp intron, encoding a protein of 202 amino acids with molecular weights of 23.5 kDa. The protein sequence shows 82.7%, 66.0%, and 41.2% identities to Sec22 proteins from *Fusarium albosuccineum*, *A. oryzae* and *S. cerevisiae*, respectively. Phylogenetic analysis revealed that the BbSec22 is most closely related to the Sec22 protein of the insect pathogenic fungus *Cordyceps militaris* (Figure 2A). Domain prediction revealed that BbSec22 contains a conserved SNARE domain and a transmembrane motif located in the C-terminus. The conserved amino acid residue in the SNARE domain is R, indicating that BbSec22 belongs to the R-SNARE superfamily (Figure 2B).

The temporal transcript pattern of *BbSEC22* in cultures of *B. bassiana* grown for 2–8 days on SDAY plates at 25 °C is illustrated in Figure 2C. As a result of qRT-PCR analysis, the transcript level of *BbSEC22* was gradually increased with the incubation time on the previous 6 days but down-regulated during the following 2 days. The transcriptional expression of *BbSEC22* on day 6 was 4.3-fold and 1.8-fold higher than that on day 2 and day 8, respectively.

### 3.3. Targeted Deletion and Complementation of BbSEC22

**Figure 3:**
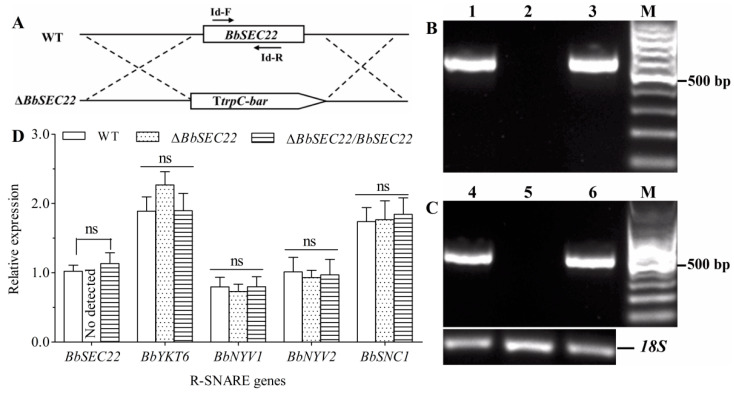
Disruption and complementation of *BbSEC22* in *B. bassiana*. (**A**) Diagram for *BbSEC22* deletion. (**B**,**C**) Identification of the deletion and complement mutants of *BbSEC22* by PCR (**B**) and RT-PCR (**C**). Lanes 1 and 4 were loaded with the samples of wild-type strain, Lanes 2 and 5 were loaded with the samples of Δ*BbSEC22*, and Lanes 3 and 6 were loaded with the samples of Δ*BbSEC22*/*BbSEC22*, respectively. The bands of the last row showed with internal reference 18S in different samples. (**D**) The relative transcript level of *BbSEC22* and other four R-SNARE genes in the 3-day-old SDAY cultures of the deletion and complementation mutants versus the wild-type strain. Error bars: standard deviation of the mean from three cDNA samples of each strain detected in qRT-PCR experiments. ns on bars denote no significant differences between samples.

The *BbSEC22* gene was disrupted by replacing it with the *bar*-vectoring cassette via double homologous recombination (Figure 3A). The complemented mutant was constructed by inserting the full expression element of *BbSEC22* with the *sur* marker through *Agrobacterium*-mediated transformation. The deletion and complementation mutants of *BbSEC22* were first identified by PCR analysis using the specific primer pairs (Table S1, Figure 3B). RT-PCR experiments using primers targeted to the *BbSEC22* cDNA sequence showed complete loss of the transcript in the deletion strain, as the targeted fragment (529 bp) of *BbSEC22* was amplified from the control strains (wild type and complemented strains) but not from the deletion mutant (Figure 3C). Targeted gene deletion and complementation were further confirmed by qRT-PCR assays. Consequently, the transcript of *BbSEC22* in the deletion mutant was undetectable but present in the control strains in qRT-PCR experiments, indicating again a success for gene deletion and complementation. Additionally, deletion of *BbSEC22* had no significant effect on the expression levels of *BbYKT6*, *BbNYV1*, *BbNYV2*, and *BbSNC1*, the other four R-SNARE genes presented in the *B. bassiana* genome (Figure 3D). The obtained deletion mutant (Δ*BbSEC22*) and complemented mutant (Δ*BbSEC22/BbSEC22*) were assayed together with the wild-type strain for possible alterations of various phenotypes as follows.

### 3.4. Effects of the BbSEC22 Deletion on Mycelial Growth

**Figure 4:**
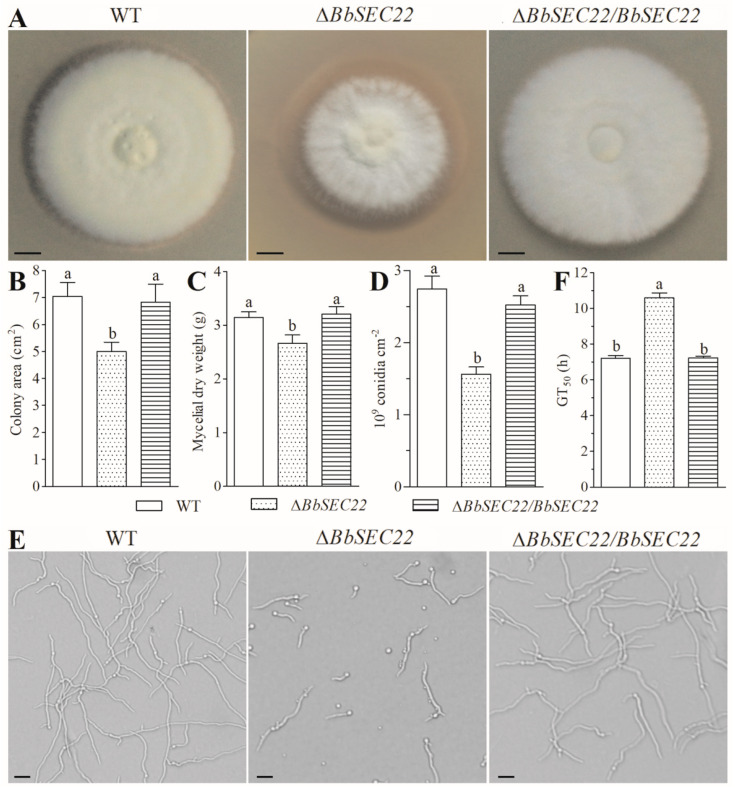
Effects of *BbSEC22* deletion on mycelial growth, sporulation capacity, and conidial germination of *B. bassiana*. (**A**) Colony morphology and (**B**) colony sizes after 8-day growth at 25 °C on the plates of SDAY. Scale bars: 5 mm. (**C**) Mycelial dry weight after 3-day growth at 25 °C in SDB media. (**D**) Conidial yields measured from the SDAY cultures grown for 8 days at 25 °C. (**E**) Conidial germlings after 12 h incubation at 25 °C on GM plates. Scale bars: 3 µm. (**F**) GT_50_ (h) estimates for the time required to achieve 50% conidial germination. Error bars: standard deviation from three repeated assays. Different letters on the bars denote significant differences in each group (Tukey’s HSD, *p* < 0.05).

The deletion of *BbSEC22* resulted in conspicuous changes in the colony morphology grown on SDAY at 25 °C. As shown in Figure 4A, the Δ*BbSEC22* mutant showed a reduced colony size, thinner mycelial edge, and more fluffy appearance in comparison to the control strains (the wild-type strain and Δ*BbSEC22/BbSEC22*). The Δ*BbSEC22* colony was 28.5% smaller than those of the control strains (~7.0 cm^2^) after 8-day growth on SDAY plates (Figure 4B). The dry weight of mycelia for the Δ*BbSEC22* mutant cultured in SDB was also significantly decreased by 15.3% when compared to that of the control strains (Figure 4C).

### 3.5. Effects of the BbSEC22 Deletion on Sporulation and Conidial Germination

Conidial yields measured from the colonies differed significantly between the Δ*BbSEC22* mutant and the control fungal strains during the incubation period. Both the wild-type strain and the complement strain started sporulation on day 3 and produced significantly more conidia than the deletion mutant on day 8. The final conidial yield of the Δ*BbSEC22* mutant was severely reduced by 43.1% when compared with that measured from the control strains (Figure 4D). Additionally, the Δ*BbSEC22* mutant exhibited an obvious lag in conidial germination (Figure 4E). In comparison to the control strains, in which the germination rate was nearly 100% at the end of 9 h, the Δ*BbSEC22* strain had a germination rate of only 78.6% after 12 h. The time (GT_50_) required for 50% germination was delayed by 3.4 h compared with the mean GT_50_ (7.2 h) for the control strains (Figure 4F).

### 3.6. Effects of the BbSEC22 Deletion on Stress Tolerance

**Figure 5:**
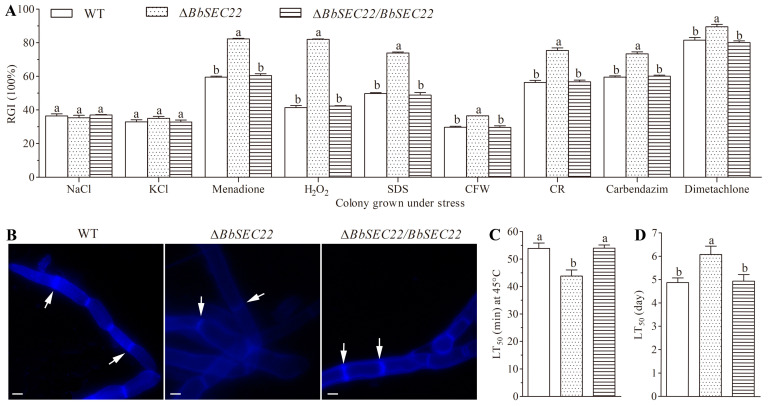
Effects of *BbSEC22* deletion on stress tolerance and insecticidal virulence of *B. bassiana*. (**A**) Relative colony growth inhibition after 7-day growth at 25 °C on the plates of 1/4 SDAY supplemented with different stress agents. CFW: calcofluor white, CR: Congo red. (**B**) Alteration of chitin distributed in cell wall based on CFW staining. The septum of mycelia was pointed by arrows. Scale bars: 3 µm. (**C**) Median lethal times (LT_50_) for conidial tolerances to heat stress at 45 °C (min). (**D**) Median lethal time (LT_50_ in day) for fungal virulence to the second-instar larvae of *S. litura*. Error bars: standard deviation from three repeated assays. Different letters on the bars denote significant differences in each group (Tukey’s HSD, *p* < 0.05).

As a result of the *BbSEC22* deletion, the capacity of mycelial stress tolerance was observed severely defective (Figure 5A). Based on the percent growth inhibition of the strains relative to unstressed controls, the Δ*BbSEC22* mutant was significantly more sensitive to oxidative stress than the control strains. The relative growth inhibition of the Δ*BbSEC22* colonies was significantly increased by 22.9% and 40.6% by adding 3 mM menadione and 40 mM H_2_O_2_ compared to the estimates from the wild type and Δ*BbSEC22/BbSEC22* strains. However, the osmotic salts (1 M NaCl or KCl) had no effect on the Δ*BbSEC22* mutant compared to its relative growth inhibition (RGI) values with the mean from the control strains. A pronounced decrease in colony size of the Δ*BbSEC22* mutant was observed when grown on plates containing cell wall perturbing agents. In the presence of 0.1% SDS, the RGI of the Δ*BbSEC22* colonies was drastically decreased by 48.6%. Colony tolerance was also significantly inhibited by CFW and CR, which caused a 23.1% and 33.8% tolerance decline, respectively. CFW staining was further used to probe the distribution of chitin, one of the main components of the fungal cell wall. In the wild type and Δ*BbSEC22/BbSEC22* strains, CFW fluorescence was mostly distributed at the septa and tips, where chitin was actively synthesized, while in Δ*BbSEC22*, fluorescence was observed brighter on the lateral wall than the septum of the hyphae (Figure 5B). Moreover, the deletion of *BbSEC22* caused significantly decreased tolerance to two fungicides, as the relative growth inhibition was increased by 19.1% and 9.8% for the Δ*BbSEC22* colonies grown on the SDAY plates supplemented with 1 μg mL^−1^ carbendazim and 0.1 mg mL^−1^ dimetachlone, respectively. The effect of high temperature on the conidial survival of the deletion mutant and control strains after 24 h germination in GB was also examined. Compared with the LT_50_s from the control strains, conidial tolerances to heat stress of 45 °C were reduced by 18.7% in Δ*BbSEC22* (Figure 5C).

### 3.7. Effects of the BbSEC22 Deletion on Pathogenicity

In the bioassays of *S. litura* larvae topically inoculated by the standardized spray of 1 mL conidial suspension, the final mortalities on day 7 were 66.7% for Δ*BbSEC22* and about 88.9% for the control strains, respectively. As a result of the modeling analyses, the LT_50_ value for the Δ*BbSEC22* mutant was 6.1 days, which was 24.5% longer than the estimate from the control strains (about 4.9 days) (Figure 5D).

### 3.8. Transcript Changes of Phenotype-Associated Genes in the BbSEC22 Deletion Mutant

**Figure 6:**
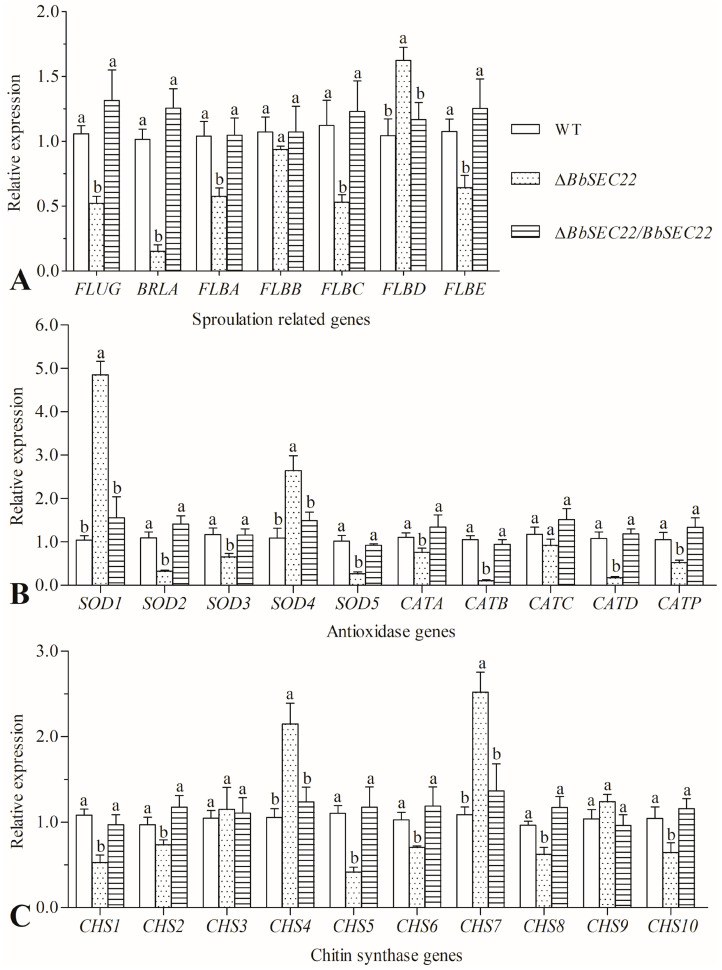
Relative transcript levels of phenotype-associated genes involved in (**A**) fungal sporulation, (**B**) oxidative stress tolerance, and (**C**) cell wall integrity maintenance in the *BbSEC22* colonies grown on SDAY plates for 3 days at 25 °C. The transcript of the target gene in the wild-type strain (WT) was used as a standard for comparison. Error bars: standard deviation from three repeated assays. Different letters on the bars denote significant differences in each group (Tukey’s HSD, *p* < 0.05).

The transcript levels of 27 genes essential for conidiation and stress tolerance to oxidation and cell wall disturbance were assessed in the total RNAs from the 3-day SDAY colonies of the deletion mutant and control strains through qRT-PCR with paired primers (Table S2). As illustrated in Figure 6A, five out of seven genes required for conidiophore development and conidiation were all down-regulated in the Δ*BbSEC22* mutant. Of those, the *FLUG* and *BRLA* were drastically suppressed by 50.6% and 85.0% compared with that in the control strains, respectively. Similar transcriptional changes were also observed for the genes involved in detoxification. Among five superoxide dismutases and five catalase-encoding genes, seven out of them were significantly suppressed by 31.3–89.7% (Figure 6B). Chitin synthases were key enzymes to synthesize chitin in the fungal cell wall. Interestingly, the results showed that exception of *CHS3*, *CHS4*, *CHS7*, and *CHS9*, six out of ten chitin synthase genes were significantly down-regulated in the Δ*BbSEC22* mutant in comparison to the control strains. For instance, the expressions of *CHS1* and *CHS5* were significantly repressed by 51.1% and 62.4% in the *BbSEC22* deletion mutant, respectively (Figure 6C). The transcript changes of the grouped genes in the deletion mutant were considerably in agreement with the phenotypic alterations in its sporulation capacity, responses to oxidants, and cell wall integrity.

## 4. Discussion

Insect fungal pathogens, including *Beauveria* spp. and *Metarhizium* spp., have been used successfully as biocontrol agents against plant-feeding and blood-sucking pests [32]. During the invasive and developmental processes of fungal entomopathogen, the fungal cells necessarily secrete a large and diverse set of cuticle-degradation enzymes such as chitinases, proteases, and lipases [43,44], soluble toxic components such as beauvericin, oosporein and destruxin [45,46], and other secreted compounds such as glycerol, trehalose and mannitol, that contribute to the pathogenicity and stress tolerance of the fungus [47,48]. Because of the potential importance of vesicle transport of these effectors secretion and the core function of SNAREs in cellular vesicle trafficking, we identified the SNARE families at the genome-wide level in *B. bassiana* and functionally characterized a conserved R-SNARE encoding gene *BbSEC22*. As presented above, BbSec22 plays important roles not only in regulating mycelial growth and conidiation but also in mediating fungal virulence and stress tolerance.

Our systematic analysis revealed the presence of 22 SNARE-encoding genes in the whole genome sequence of *B. bassiana* and will facilitate theidentification of SNARE orthologs in other filamentous fungi. Most of the SNAREs in *S. cerevisiae* could find their counterparts in *B. bassiana*, except forthe homologs of Use1 and Spo20 [4]. The number of *B. bassiana* SNAREs was comparable with that in other filamentous fungi, such as 20 SNAREs in *N. crassa*, 21 in *F. graminearum*, and 22 in *V. dahlia* but less than that from *Phytophthora* species, which possessing 34–37 SNAREs [2,5,6]. Although *B. bassiana* SNAREs showed relatively low sequence similarity to their counterparts in yeast, most of them still share the essential domain structures such as the conserved SNARE motif and the C-terminal transmembrane regions, and hence, it is highly conceivable that they act as SNAREs in *B. bassiana*. Typically, an arginine (R) or glutamine (Q) residue was conservatively located at the zero layer in the four-helical bundles of the SNARE domain, which was considered to be essential to neutralize the electric charge for the SNARE complex. However, residue substitutions were detected in a few *B. bassiana* SNAREs. For instance, the essential residue of BbSyn8 was a histidine (H) and BbBet1 was a serine (S), indicating that they might not act as conventional SNAREs [5]. These substitution events were also observed in protein structures of SNAREs from the filamentous fungus *A. oryzae*. Some Qa-SNAREs have two or more homologs in the genomic sequences of several fungi, such as Sso1 and Sso2 in *S. cerevisiae* that are functionally redundant [49], GzSyn1 and GzSyn2 in *Gibberella zeae* that are functionally different [22]. Similarly, three homologs BbSso1, BbSso2a, and BbSso2b were found in *B. bassiana*. BbSso2a and BbSso2b shared high sequence identities with each other and might be functionally redundant, whereas both of them showed low sequence similarity with BbSso1, suggesting that they might play a different role in fungi between 1 and 2 types. Besides BbSec22, the *B. bassiana* harbors other four R-type SNARE proteins (BbYkt6, BbNyv1, BbNyv2, and BbSnc1). Of those, BbNyv1 and BbNyv2 possess all three similar conserved domains with Bbsec22. Nevertheless, BbSec22 exhibited low sequence similarity (less than 25%) with these two R-type SNARE proteins. In addition, no obvious expression changes of these four R-SNARE genes were detected in the absence of *BbSEC22*. These results highly indicated that diverse roles were presented among the R-SNAREs in *B. bassiana*.

Deletion of *BbSEC22* in *B. bassiana* resulted in a plethora of phenotypic defects, from mycelial growth to asexual sporulation. The Δ*BbSEC22* mutant exhibited reduced colony size, thinner mycelial edge, and more fluffy appearance, in agreement with the observations in the *SEC22*-deletion mutants of *M. oryzae*, *F. graminearum* and *A. oligospora* [19,24,29]. Hyphal growth of filamentous fungi requires large amounts of metabolites that are usually delivered by vesicle trafficking [2]. The reduced growth rate and abnormal hyphal morphology of Δ*BbSEC22* observed here indicated that BbSec22 might be involved in the trafficking processes required for fungal growth and development in *B. bassiana*. Apart from the involvement in the regulation of mycelial growth, Sec22 in *M. oryzae*, *F. graminearum*, and *A. oligospora* were also proven to be essential for asexual sporulation [19,24,29]. Similarly, the *BbSEC22* deletion mutant reduced the conidial production as well as delayed the conidial germination. Significant impairment of mycelial growth could probably be responsible for the decreased sporulation capacity. Another factor that contributed to the conidiation defect could be the significant down-regulation of several sporulation-required genes, such as *FLUG* and *BRLA* that are essential for the conidiation of *B. bassiana* [50,51]. Like hyphal growth, conidia differentiation of filamentous fungi in particular requires the transfer of many matrix components within the fungal mycelia [52]. It is conceivable that *BbSEC22* might be involved in the trafficking processes required for conidia maturation and germination in *B. bassiana*.

BbSec22 was also proven to be involved in cellular responses to multiple abiotic stresses in *B. bassiana*. Deletion of *BbSEC22* resulted in increased sensitivity to extracellular oxidative stress (exposed to menadione or H_2_O_2_) during colony growth, in accordance with those observed from the *Sec22-*deletion mutants of other filamentous fungi [24,29]. Such defects in the antioxidant defense are likely associated with the transcription repression of several antioxidase-encoding genes, such as SOD-encoding genes *SOD1*, *SOD2*, and catalase-encoding gene *CATP* that crucial for the oxidative tolerance of *B. bassiana* [53]. Another possibility is that BbSec22 is required for the recruitment of these ROS (reactive oxygen species) scavenging enzymes to their proper destination. The Δ*BbSEC22* mutant also exhibited higher sensitivity in cell tolerance to cell wall perturbing stress (treated with CFW and CR), coupled with an abnormal distribution of chitin, a key constituent of the fungal cell wall. It is well-documented that fungal chitin biosynthesis depends on the activity of chitin synthase [54,55]. In the present study, many chitin synthase encoding genes were significantly down-regulated in their transcriptional levels in the *BbSEC22* deletion mutant. It has also been proved that cell wall synthesis requires the endocytic uptake and recycling of cell wall proteins [56]. Deletion of *SEC22* in *S. cerevisiae* and *M. oryzae* caused endocytosis defects and reduced re-uptake of chitin synthases which impaired fungal growth [24,57]. In *F. graminearum*, deletion of *FgSEC22* led to increased sensitivity to osmotic agents [29]. However, the cell tolerance to hyperosmotic stress was not affected by the *BbSEC22* deletion in this study. Moreover, the Δ*BbSEC22* mutant became more sensitive to carbendazim and dimetachlone during colony growth, indicating that BbSec22 was also functionally involved in the fungicide resistance in *B. bassiana*.

Several SNARE proteins have been proven to be involved in the fungal pathogenicity of plant pathogenic fungi, such as Syn8, Sec22, and Vam7 in *M. oryzae* and *F. graminearum* [8,20,24,25,26,29]. Consistently, in the present study, insecticidal virulence in the larvae of *S. litura* was found to be severely reduced in the Δ*BbSEC22* mutant. In view of seriously impaired hyphal growth and delayed conidia germination in Δ*BbSEC22* mutant, we could not eliminate that the reduced insecticidal activity might be due to these growth defects. However, in recent, several studies on fungal pathogens have provided evidence that processes required for secretion are essential for pathogenicity by controlling morphogenesis and delivery of important virulence-associated proteins [29,30,58]. Thus, it is possible that deletion of *BbSEC22* resulted in impairment of secretion pathways of virulence factors, causing the attenuation of virulence. For example, protein toxins, cell wall degrading enzymes, extracellular proteases, stress-response-related enzymes, or some important signal receptors are key virulence factors in *B. bassiana* [38,45,59,60]. The direct or indirect relationship between the secretion of these factors and the SNAREs needs to be further studied in detail.

Overall, our results indicated that *BbSEC22* encodes a putative SNARE that is classified into the R-SNAREs group by conserving sequence alignment. The *BbSEC22* deletion mutant showed a fluffy appearance in mycelial growth, an obvious lag in conidial germination, significantly increased sensitivity to oxidative stress and cell wall perturbing agents, a remarkable loss of conidiation, and a significant decrease in conidial virulence to *S. litura* larvae. Such defects are likely associated with the transcription repression of several defense-related encoding genes.

## 5. Conclusions

The results presented here indicated that R-SNARE protein BbSec22 plays crucial roles in the regulation of vegetative growth, asexual sporulation, conidial germination, multi-stress tolerance, and insecticidal virulence in *B. bassiana*. Future works should focus on characterizing other SNAREs and target-proteins delivered by these SNARE complexes, which may easier to be illuminate the relationship between fungal development and vesicle trafficking mediated by SNAREs and potentially lead to new means to produce *B. bassiana* new strains with great potential in pest biocontrol.

## Figures and Tables

**Table 1:** Putative SNAREs in *B. bassiana* and their homologs in *S. cerevisiae*.

Type	Name	Protein_ID	aa	Gene_ID	Gene (bp)	CDS (bp) ^a^	Intron	Zero Layer Residue ^b^	Homologs in *S. cerevisiae*
Qa-SNARE	BbUfe1	EJP69482	352	BBA_01447	1179	1059	2	Q	Ufe1
	BbSed5	EJP63375	320	BBA_07769	1074	963	2	Q	Sed5
	BbTlg2	EJP63918	344	BBA_07242	1161	1035	2	Q	Tlg2
	BbSso1	EJP64917	326	BBA_06092	1377	981	2	Q	Sso1
	BbSso2a	EJP63095	411	BBA_07900	1441	1236	2	Q	Sso2
	BbSso2b	EJP61869	220	BBA_09206	775	663	1	Q	Sso2
	BbPep12	EJP66927	263	BBA_04220	849	792	1	Q	Pep12
Qb-SNARE	BbSec20	EJP61319	429	BBA_09714	1390	1290	1	Q	Sec20
	BbBos1	EJP65062	256	BBA_05832	908	771	1	Q	Bos1
	BbGos1	EJP69616	228	BBA_01581	830	687	2	Q	Gos1
	BbVti1	EJP64999	203	BBA_06174	742	612	2	Q	Vti1
Qc-SNARE	BbSft1	EJP63515	97	BBA_07441	406	294	1	D	Sft1
	BbBet1	EJP67882	244	BBA_02778	1754	1230	2	S	Bet1
	BbTlg1	EJP64309	239	BBA_06691	779	720	1	Q	Tlg1
	BbSec9	EJP65468	427	BBA_05799	1336	1284	1	Q	Sec9
	BbSyn8	EJP60748	261	BBA_10304	860	786	1	H	Syn8
	BbVam7	EJP66712	365	BBA_04005	1098	1098	0	Q	Vam7
R-SNARE	BbSec22	EJP64509	202	BBA_06503	696	609	1	R	Sec22
	BbYkt6	EJP70865	197	BBA_00495	984	594	4	R	Ykt6
	BbNyv1	EJP61800	234	BBA_09220	799	705	1	R	Nyv1
	BbNyv2	EJP61468	230	BBA_09604	819	693	2	R	Nyv2
	BbSnc1	EJP66249	138	BBA_04742	884	417	1	R	Snc1

^a^ CDS denotes the full length of the cDNA sequence. ^b^ Bold and underlined letters indicate unconventional residues at the zero layers in the four-helical bundles of the SNARE domain.

## Data Availability

Data are contained within the article and Supplementary Materials.

## Supplementary Materials

The following supporting information can be downloaded at: https://www.mdpi.com/article/10.3390/jof10060393/s1, Table S1: The primers designed for gene cloning, expression, deletion, complementation and mutant identification. Table S2: Paired primers used in qRT-PCR for the transcript levels of the phenotype-associated genes.
