# Supplementary material for: Genome-Wide Identification of SNARE Family Genes and Functional Characterization of an R-SNARE Gene BbSEC22 in a Fungal Insect Pathogen Beauveria bassiana
Source: J Fungi (Basel). 2024 May 31;10(6):393. doi: 10.3390/jof10060393 (PMC11204939; doi:10.3390/jof10060393)
Supplement: Supplementary file 1 [file jof-10-00393-s001.zip › Table S1.pdf]

Table S1. The primers designed for gene cloning, expression, deletion, complementation and mutant identification.

Primers	Sequences (5′-3′) *	Purpose
Sec22-F	ATGCTCTGCGCCTCAGTCGACGACG	Cloning *BbSec22*
Sec22-R	TTAGAAAATTCGCCAGTAGATGAAG	
Sec22q-F	CGAGATGAGCAGCAGGTTGAG	qRT-PCR for R-SNAREs
Sec22q-R	AATTCGCCAGTAGATGAAGAGGAG	
Ykt6 q-F	GCGAAACAACTATGGCGAATT	
Ykt6 q-F	ACCTTGCTGAGGACCTGATGG	
Nyv1q-F	CGTGCCCTTTGGCTACCTTGT	
Nyv1q-R	AGTGTCTTGAGCGTGCCGTTG	
Nyv2q-F	CACCCAAATCACCCATCTGCC	
Nyv2q-R	GCTATACTCGACCATGAGCGTCTT	
Snc1q-F	CAGGCTCTGCAAGCTCAAATCG	
Snc1q-R	CGCATCTTCATGTCCTTCCACC	
18S-F	TGGTTTCTAGGACCGCCGTAA	
18S-R	CCTTGGCAAATGCTTTCGC	
Sec22up-F	CCCAAGCTTCGCTGTTGAAATGTGCTCTGTCGGT	Deleting *BbSec22*
Sec22up-R	CGGCGAGCTCATGACGGCATGTTTCAATGATGGGA	
Sec22dn-F	GGAAGATCTGAACGGCGTACGGAAACGAATGCAC	
Sec22dn-R	CTAGACTAGTGAATGAACGGGAAACTCATGGATGG	
Sec22c-F	GGGGACAAGTTTGTACAAAAAAGCAGGCTGTGGT<br>GTAACCTTGCGATGCTCAG	Rescuing *BbSec22*
Sec22c-R	GGGGACCACTTTGTACAAGAAAGCTGGGTCCCTG<br>GACCACTTTCTTGTTTCTTC	
Id-F	AGAACCAGGTCCGCCAAATC	PCR, RT-PCR for *BbSec22*
Id-R	GAGGAGAATAATGAAGCCCAGCAC	

* The introduced cleavage sites of the enzymes *Hin*dIII, *Sac*I, *Bgl*II and *Spe*I are underlined.
