# Supplementary material for: Genome-Wide Identification of SNARE Family Genes and Functional Characterization of an R-SNARE Gene BbSEC22 in a Fungal Insect Pathogen Beauveria bassiana
Source: J Fungi (Basel). 2024 May 31;10(6):393. doi: 10.3390/jof10060393 (PMC11204939; doi:10.3390/jof10060393)
Supplement: Supplementary file 1 [file jof-10-00393-s001.zip › Table S2.pdf]

Table S2. Paired primers used in qRT-PCR for the transcript levels of the phenotype-associated genes.

Gene	Gene_ID	Annotation	Sequences (5′-3′) of paired primers (sense/antisense)
**Involved in fungal sporulation**			
FLBA	BBA_02968	Conidiation transcription factor A	TCTCTTCTCTACCTTGATTGT / AATGTGGTGGTTGTTGTG
F LBB	BBA_06988	bZIP-type transcription factor	GCTGTTGGACCTGAGTAA / AATCCGTAACATCTGACCTT
FLBC	BBA_03181	Conidiation transcription factor C	CTCCTACTATGGCGACTC / GTGCTTGATGAGACCTTG
FLBD	BBA_07259	Conidiation transcription factor D	GCTGCCTCCAATAAGAAC / CTGTGCGTATCGTTATCG
FLBE	BBA_01716	Conidiation transcription factor E	CAGACGATGAGACAGAGA / GGGCTTTATATGCGAGTAG
FLUG	BBA_04942	Developmental protein FluG	CCATCAGGATACATCGTCTT / AGATTCATAGTCTCGCTCAA
BRLA	BBA_07544	Zinc finger transcription factor protein	GACCAGTTCAACAGACAAG / CAGTAATCTTCGTGCTTCTC
**Involved in antioxidant responses**			
SOD1	BBA_02311	Cytosolic Cu/Zn superoxide dismutase	GCCGTACCGTCGTTGTCCAC / TCCAGCGTTGCCAGTCTTGAG
SOD2	BBA_09706	Cytosolic Mn superoxide dismutase	CCTACCTTGCCCTACGCCTAC / GTGGTGCTTGGTGTGGTGAAG
SOD3	BBA_09382	Mitochondrial Mn superoxide dismutase	CAGGCTCCTCTTCTCAACTTCC / TCCTCGGTGATGGCAGTGG
SOD4	BBA_04317	Superoxide dismutase	ACCTGACGCACGACTTCTCC / CGATGGTCTTGGTGTCCTTATCC
SOD5	BBA_01984	Superoxide dismutase	GCTATCGGCGGCAACTGTAC / GTCGGTGAACTCGGTGATGAAC
CATA	BBA_06186	Catalase A	GAAAGCCGCGCAAGTGAAAG / TCTCTGGCAAAGACATCCTCAAG
CATB	BBA_05603	Catalase B	GAAGACGCCCATGTTTGTTCG / AAAGTTGCCCTCATCGGTATAGC
CATC	BBA_09338	Catalase C	TGTACTGGGGCTCCGAACC / ATGAGACCTGTGTAGCGTTAGC
CATD	BBA_09109	Catalase D	TGCTGGACGATGTGTCTGAC / CACGCACCGTATCGCTAGAG
CATP	BBA_09760	Catalase P	GCGCTCGCAGTGACTGTAC / CTAGCACGGCCCTGTATAATGG
**Involved in cell wall integrity**			
CHS1	BBA_03590	Chitin synthase	GACGGCACAGGCAAGACAG / GGTGAGCATCCAGACGAGAAC
CHS2	BBA_03236	Chitin synthase	GAGACTGGCTGGCACAAGATTG / GGCGATACCGTGTTGGAAGAC
CHS3	BBA_04667	Chitin synthase	TCGCTGGCTCAACGGTTCC / TGACAGTGGTGGTGAGGTAGTAAG
CHS4	BBA_06846	Chitin synthase	GGAGAATCAGTGCTACCTCAATGG / TTGCCCGTTGTGCTGCTATG
CHS5	BBA_02360	Chitin synthase	GGTCCGCACGCACAAGATG / CGCACGCCGTCCACAATC
CHS6	BBA_07346	Chitin synthase	GCCGCCGTCAATGCTCTC / TCTTCTGCTGGGTCCTCGTAG
CHS7	BBA_03793	Chitin synthase	CTCGGCGTAATGATGGTCTACAAG / GCACGGCGATCCTCCTCAG
CHS8	BBA_06859	Chitin synthase	CTCCTTGCCGACTCATCTTCAG / CTTCCCTCTTTGCCGCCATC
CHS9	BBA_08044	Chitin synthase	CCAGTATCACCACCAGCAAGAC / CTGAGTAGACCGAGTACGAGGAG
CHS10	BBA_08549	Chitin synthase	TGTTCTATTCGCCATCGGTCAAG / AGACAGCAACAACGGACAAGAG
